# Lhermitte’s sign

**DOI:** 10.1590/S1516-31802010000100010

**Published:** 2010-01-07

**Authors:** Viroj Wiwanitkit

**Affiliations:** 1 MD, PhD. Professor, Wiwanitkit House, Bangkhae, Bangkok, Thailand.

## Dear Editor,

I read the recent paper by Teive et al. with great interest.^1^ They mentioned that the case report was a rare case of vitamin B 12 deficiency firstly presenting with Lhermitte’s sign. This is still questionable. Indeed, before any neurological manifestation in cases of vitamin B12 deficiency, anemia development may be seen. Such anemia may present several signs and symptoms. In addition, Lhermitte’s sign can be seen in other disorders that may be observed among the elderly, and this was not completely ruled out in the report by Teive et al.^1^ The best examples of such disorders are occulted malignancy and multiple sclerosis.^2,3^
